# Tunable quasiparticle trapping in Meissner and vortex states of mesoscopic superconductors

**DOI:** 10.1038/ncomms10977

**Published:** 2016-03-16

**Authors:** M. Taupin, I. M. Khaymovich, M. Meschke, A. S. Mel'nikov, J. P. Pekola

**Affiliations:** 1 Low Temperature Laboratory, Department of Applied Physics, Aalto University School of Science, P.O. Box 13500, FI-00076 Aalto, Finland; 2 Institute for Physics of Microstructures, Russian Academy of Sciences, GSP-105, 603950 Nizhni Novgorod, Russia; 3 Lobachevsky State University of Nizhni Novgorod, 23 Prospekt Gagarina, 603950 Nizhni Novgorod, Russia

## Abstract

Nowadays, superconductors serve in numerous applications, from high-field magnets to ultrasensitive detectors of radiation. Mesoscopic superconducting devices, referring to those with nanoscale dimensions, are in a special position as they are easily driven out of equilibrium under typical operating conditions. The out-of-equilibrium superconductors are characterized by non-equilibrium quasiparticles. These extra excitations can compromise the performance of mesoscopic devices by introducing, for example, leakage currents or decreased coherence time in quantum devices. By applying an external magnetic field, one can conveniently suppress or redistribute the population of excess quasiparticles. In this article, we present an experimental demonstration and a theoretical analysis of such effective control of quasiparticles, resulting in electron cooling both in the Meissner and vortex states of a mesoscopic superconductor. We introduce a theoretical model of quasiparticle dynamics, which is in quantitative agreement with the experimental data.

## Abstract (web summary)

Excessive excitation induced by overheating may deteriorate the resistance-free operation of superconductor-based devices. Here, Taupin *et al*. propose an effective control of excess quasiparticles and their spatial distribution in a mesoscopic superconducting disc by applying a small magnetic field.

## Graphical abstract

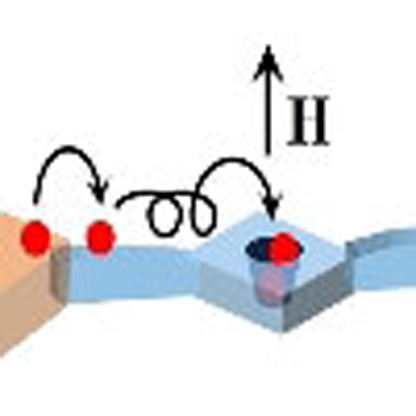

The presence of excess quasiparticles (QPs) is often characterized by an effective electron temperature *T* that exceeds the temperature of the phonon bath *T*_0_. The resulting overheating is known to be the origin of such effects as decoherence in qubit systems^1^,^2^,^3^, decrease of the quality factor of superconducting resonators^4^,^5^, the excess current in single-electron turnstiles^6^, and low efficiency of electronic cooling in normal metal (N)–insulator (I)–superconductor (S) junctions^7^,^8^. In short, overheating is a major factor limiting the performance of S mesoscopic devices. More than the overall quasiparticle number *N*_qp_, the critical parameter is the location of these excess quasiparticles. For instance, for tunnel junction circuits, it is crucial to avoid the quasiparticles in a superconductor nearby the junction, while the extra quasiparticles located further away are of less concern. To suppress overheating in a superconductor one aims at lowering the generation of extra quasiparticles in the whole superconductor using proper electromagnetic shielding of the device, and decreasing the quasiparticle density by introducing quasiparticle traps (see for example, refs 9, 10), by optimizing the device geometry^6^,^11^, or by cooling using the tunnel junction to another superconductor with a larger gap^12^,^13^,^14^. The second method allows one to move quasiparticles away from critical locations and relax them. Quasiparticle traps have become an important element in designing devices for mesoscopic physics and metrology.

The most common ones among different types of quasiparticle traps are normal metal sinks^15^,^16^,^17^, Andreev bound states in weak links^18^, special S gap engineering^19^,^20^,^21^,^22^,^23^ and non-uniform superconducting states induced by an external magnetic field^24^,^25^,^26^,^27^. Here we focus on the magnetic field controlled trapping, a method which has a number of advantages. The regions with the reduced gap in this case are of the same material as the rest of the device and therefore match perfectly the S parts without barriers or interface potentials. Besides, magnetic field gives the possibility to make tunable traps allowing, for example, the modulation of a resonator quality factor, needed for giant pulse formation (or Q-switching) in pulse lasing (see, for example, a book^28^). The controllable use of such traps in various applications mentioned above assumes, certainly, understanding of their cooling capacities, which is necessary to optimize the designing of the particular trap configurations for different mesoscopic devices.

Our work aims to the solution of this ambitious and important problem focusing on both experimental and theoretical study of individual traps, which appear in the Meissner and vortex states. To build a quantitative model of these traps we choose to verify it by the experimental measurements of the characteristics of non-equilibrium quasiparticle distributions in a mesoscopic S island (Al) in a single-electron transistor set-up with normal metal (Cu) leads. This particular device appears to provide a very convenient way to tune both the trap pattern applying an external magnetic field to the S island and the number of non-equilibrium quasiparticles injected in the island in the Coulomb blockade conditions by operating it as a turnstile of single electrons^29^. The turnstile operation frequency *f* of the gate voltage modulation controls quasiparticle injection rate. This set-up allows one to probe single quasiparticle excitations in the superconducting dot by measuring the average turnstile current under pumping conditions^6^ (ideally this current equals *ef*) and to independently control the vortex number in the superconductor^30^. The resulting trap model has perfectly proved its validity and efficiency in this set-up which can be used in future applications.

## Results

### Qualitative description

**Figure 1:**
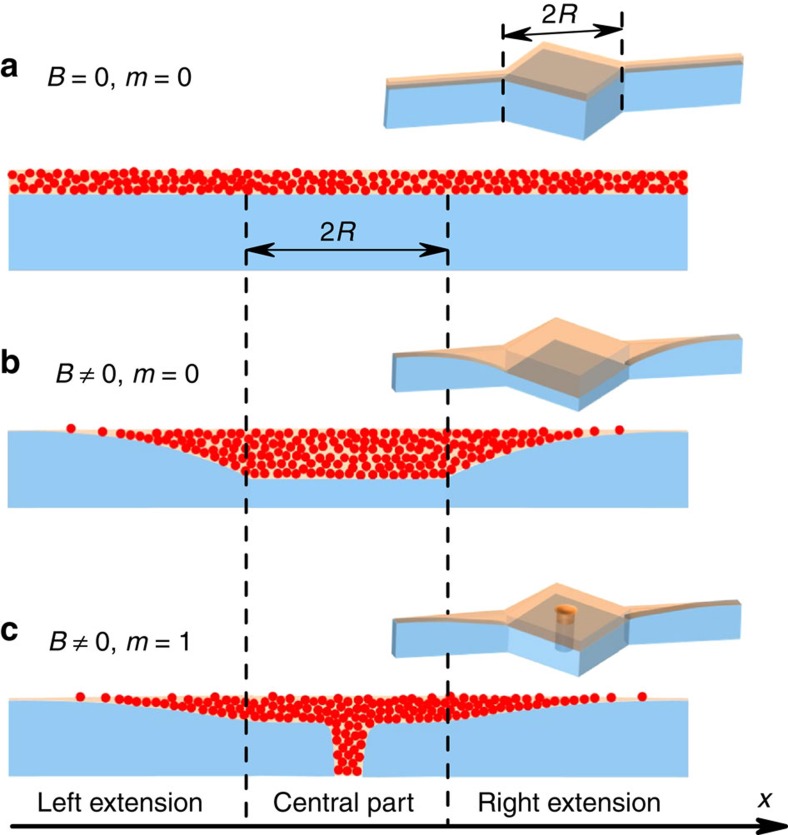
QP density and gap distributions in a S disc. The S gap *E*_g_(*B*, *x*) in a S disc with narrow extensions (Sample A) is represented by the height of the blue volume, while the QP density *n*_qp_(*x*) is shown by red circles; *m* is the vorticity of the island, *B* is the magnetic field acting on the sample. The wide central part of a Sample A of size 2*R* is limited by vertical dashed lines, the narrow extensions are located on the sides. (**a**) Uniform zero magnetic field state; (**b**) Meissner state with reduced *E*_g_(*B*, *x*) in the central part at small fields in a vortex free state; (**c**) Single-vortex state with smaller gap reduction outside the vortex core than in **b**. The 3D schematics depict the corresponding *E*_g_(*B*, *x*) (in blue) and *n*_qp_(*x*) (in orange semitransparent).

We illustrate the key idea of quasiparticle redistribution by Fig. 1 in an S island with a large central part and two narrow extensions, called Sample A. In the absence of magnetic field acting on the sample, *B*=0, the quasiparticle density *n*_qp_ is nearly uniform in the S island with constant gap *E*_g_(*x*)=Δ_0_, provided the heat diffusion length 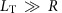 is large compared to the size of the central part *R* (see Fig. 1a). A small perpendicular magnetic field, typically few millitesla (mT), which induces Meissner screening currents flowing along the superconductor edges reduces the gap *E*_g_(*x*) mostly in the wide central part of the island but not in the narrow extensions near the junctions^31^. Due to this non-uniform gap potential *E*_g_(*x*), quasiparticles illustrated by red circles are redistributed so that their density is small at the junctions (see Fig. 1b). However, the total quasiparticle number is larger than that at *B*=0 due to its exponential dependence 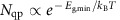 on the minimal gap 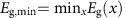 over the island, where *k*_B_ is the Boltzmann constant. A vortex in the island leads to further quasiparticle redistribution because it plays a role of a quasiparticle potential well containing a lot of quasiparticles as shown in Fig. 1c. Despite its simplicity, the theoretical model that we present below yields a quantitative fit to the experimental data on the magnetic field and frequency dependencies of the pumping current, and thus to the quasiparticle distribution, rendering the turnstile an efficient probe of quasiparticle dynamics and relaxation.

### DC measurements of the S gap

**Figure 2:**
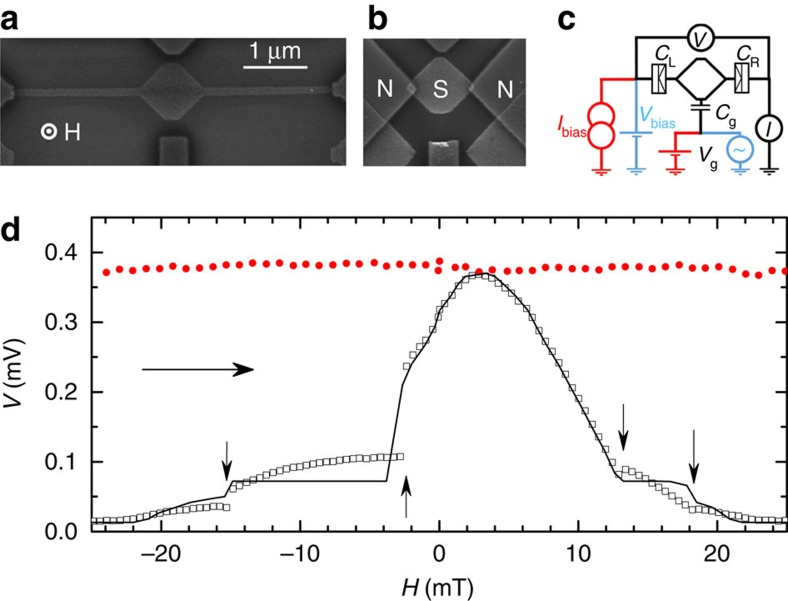
Layouts of the samples and results of d.c. measurements. Electron micrographs of (**a**) Sample A and (**b**) Sample B. (**c**) Schematic picture of the device with its electrical connections where the red and black lines correspond to the d.c. measurements, while blue and black ones to the pump measurements. (**d**) Evolution of the voltage at a fixed bias current *I*_bias_=10 pA at the gate voltage *C*_g_*V*_g_/*e*=*n*_g_=0.5 suppressing the Coulomb energy with the magnetic field for Sample A (red filled circles) and Sample B (black open squares). The vertical arrows correspond to the applied field values at which the vorticity *m* increases step by step by one from −2 to 2 as the field is swept from −25 to 25 mT. The horizontal arrow shows the direction of the field sweep. The solid black line is the theoretical result for this measurement. The experimental uncertainty has been estimated as ∼3 μV from the noise of the amplifiers.

To probe the magnetic field induced changes in the gap of a S disc, we first measure a more basic structure, which we call Sample B (see Fig. 2b). It is formed of a S disc, mimicking the central part of Sample A (Fig. 2a), directly connected via tunnel junctions to normal leads at its edges. Measuring electron transport through the disc while applying perpendicular magnetic field *H* allows us to access the field dependence of the gap value *E*_g_(*H*) at the edge of the disc and to control the vortex state. This way we can determine the critical fields for transitions between states with different vorticities *m* via simple d.c. transport measurements (similar approach as in ref. 30). We carried out current biased d.c. measurements at a gate voltage that suppresses the Coulomb energy (for the electrical configuration, see red and black lines in Fig. 2c). The experiments have been performed at a bath temperature of *T*_0_∼60 mK (well below the S gap Δ_0_ at *B*=0 and the Coulomb energy *E*_C_=*e*^2^/(2*C*), where *C* is the total capacitance of the island). Note that *B* is the actual field seen by the sample, while the applied magnetic field *H* differs from later due to some screening by the sample holder used for shielding the sample from the environment (see Supplementary Note 1).

The d.c. drain–source voltage *V* measured versus the magnetic field *H*, swept from −25 to 25 mT, is shown for Sample A (filled circles) and Sample B (open squares) in Fig. 2d at a fixed current of *I*_bias_=10 pA through the device. In general larger voltage corresponds to larger gap *E*_g_(*H*) and vice versa. The sample parameters *E*_C_, Δ_0_, and a total normal state resistance across the two junctions *R*_T_, have been extracted from *IV* measurements at zero magnetic field. For the Sample B, starting from −25 mT, the value of the voltage is small: the island is close to its normal state. The gap increases when decreasing the absolute value of the field till the maximum value reached at +2.5 mT with two intermediate step-like anomalies at 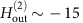 mT and 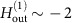 mT, corresponding to the exit of vortices, the first one from two-vortex state to one-vortex state, and the second one from one-vortex state to a vortex free state, respectively. Increasing *H* further to positive values from 2.5 to 25 mT leads to decrease of the gap again, with two knee-like anomalies at critical-field values 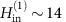 mT and 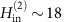 mT corresponding to the entry of the first and the second vortex, respectively. A minor distortion of the applied field (the offset in the applied field δ*H*≈2.5 mT corresponding to the maximal *V*(*H*) value and asymmetry of *V*(*H*) in the Meissner state) is caused by the sample holder, and was corrected to theoretical curves only by applying the magnetization curve *B*(*H*), with *B* the field acting on the sample, measured separately (Supplementary Note 1). Note that the magnetic field *B* acting on the sample itself, is zero at the maximal *V*(*B*=0) and corresponds to the symmetric *V*(*B*)=*V*(−*B*) in the Meissner state. The central part of Sample A has nearly the same shape and size as Sample B; thus one can expect the critical fields of these samples to be close to each other. The anomalies are absent in Sample A, as the gap near the tunnel junctions is only weakly affected by *H* in the presented range.

### Theoretical analysis of DC data

For the theoretical analysis of the above experimental data we simplify the standard Usadel model taking into account that the size of the central part *R* of the measured samples is small compared with the characteristic length scale of the Green's functions outside the vortex core regions (Supplementary Note 2 for details). Such approximation leads to the Usadel equation for the normal (cos *θ*) and anomalous (−*i* sin *θ*) Green functions


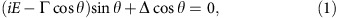


with the effective depairing parameter 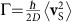 expressed through the superfluid velocity 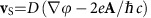 and averaged 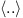 over the sample volume (over the central part of Sample A) with the excluded vortex core regions. Here 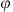 is the S order parameter phase, **A** is the vector potential determined by the magnetic field *B* acting on the sample, and *D* is the diffusion coefficient. The component of **v**_S_ perpendicular to the sample boundary and to the boundaries of vortex cores should be zero. Similarly to the previous works^19^,^20^,^24^,^25^,^26^, the vortex cores are assumed to be normal metal cylinders of the radius *r*_v_ of the order coherence length 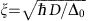, that is, *θ*=0 inside the cores. The sample size 2*R*∼1 μm is also smaller than the effective screening length *λ*_eff_∼*λ*^2^/*d*_S_∼2.6 μm, therefore we expect uniform field distribution in the island. Here *λ*∼230 nm (ref. 24) is a typical bulk penetration depth and *d*_S_≃20 nm is the thickness of the aluminium disc.

Solution of the Usadel equation gives us the standard expression for the hard gap *E*_g_ in the density of states and for the order parameter Δ as functions of Γ (see refs 32, 33, 34, 35 or Supplementary Note 2). We made a fit of the field dependence of the voltage *V*(*B*) at fixed currents *I*_bias_ using standard expressions for the current-voltage characteristic of a tunnel junction (see Supplementary Note 3 for details) and of the depairing parameter


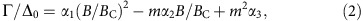


taking into account that the vector potential **A** in the superfluid velocity 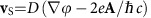 is proportional to magnetic field *B* while the S phase distribution 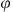 is determined by vortex sources. Here *α*_*i*_ are numerical fitting parameters, *B*_C_ denotes the field value of the first vortex entry and *m* is the total vorticity. The estimate 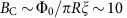 mT based on *ξ*≈100 nm and *R*≈0.5 μm is rather close to the value 
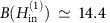
 mT from our d.c. measurements. Here Φ_0_=*h*/2*e* is the flux quantum. More accurate estimates of *B*_C_ can be done numerically, for example, within the Ginzburg–Landau approach for a concrete sample geometry^36^,^37^. According to ref. 34, the parameter *α*_1_ determines the critical value of Γ/Δ_0_ for the first vortex to enter for the Usadel equation with homogeneous **v**_S_ in a narrow strip should be 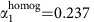, while the parameters *α*_2_ and *α*_3_ depend on the vortex configuration in the sample. The best fits to the experimental data are obtained with *α*_1_=0.38, *α*_2_=0.438, and *α*_3_=0.266, where we take 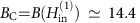 mT from experimental data. Parameter *α*_1_ for a rectangular sample is expected to be a bit larger than its value 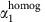 in a narrow strip^34^. In the fitting we assume that both jump-like and knee-like anomalies in the *V*(*H*) are associated with the change of vorticity^30^ and verify this applying the same parameters to *V*(*H*) with different values of *I*_bias_ (Supplementary Note 3). The S gap in the narrow extensions of Sample A shown in Fig. 2d is close to its zero-field value Δ_0_ up to ∼30 mT with few % accuracy as the depairing parameter in this case Γ/Δ_0_=(*πξwB*/Φ_0_)^2^/6 is small^35^. Here *w*≃130 nm is the width of the extensions.

### Pumping measurements

As expected from the d.c. measurements, the magnetic field does not improve the electronic pumping on Sample B (Supplementary Note 4). We thus focus only on the pumping measurements on Sample A, which has the highly non-uniform distribution of the gap (Fig. 1) under magnetic field. To probe the magnetic field dependence of non-equilibrium quasiparticle states, we measure the current *I* in turnstile mode averaged over the period of the drive 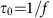 (ref. 29). We apply a fixed bias voltage *V*_bias_=100 μV and sinusoidal gate voltage 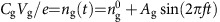 through the capacitor *C*_g_ with variable amplitude *A*_g_.

**Figure 3:**
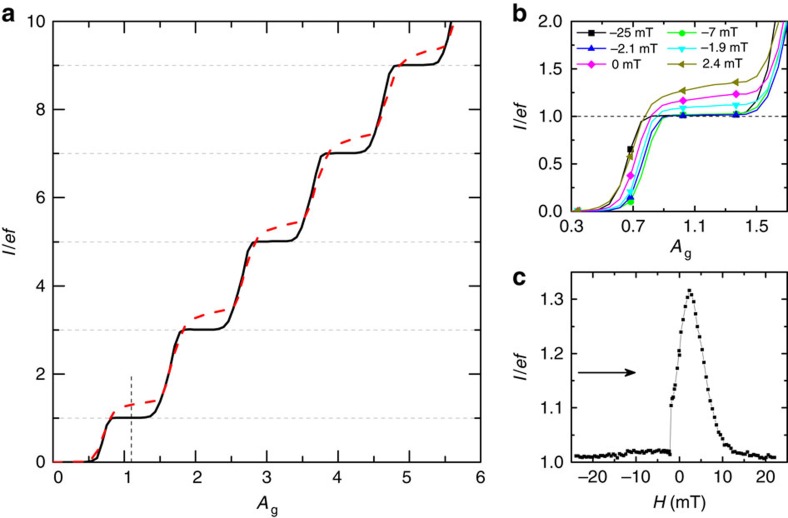
Turnstile current driven by an a.c. gate. (**a**) The pumping current *I* normalized by *ef* at the operating frequency *f*=5 MHz, the bias voltage *V*_bias_=100 μV, and at the gate offset 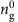
=0.5 versus the normalized gate amplitude *A*_g_ at applied field *H*=2.4 mT (*B*=0) (red dashed curve) and at *H*=−25 mT (black solid curve). The horizontal dashed lines correspond to the expected current quantization *I*=*nef*; (**b**) the zoom up of the first plateau at several applied field values between −25 and 2.4 mT; (**c**) the evolution of the current along the vertical dashed line in **a** versus the field *H* varying from −25 to 25 mT; the sweep direction is shown by the horizontal arrow. In the further measurements we fixed the amplitude to the value shown by the vertical dashed line in **a**. The experimental uncertainty has been estimated from the noise of the amplifiers as ∼10 fA, corresponding to 0.0125 *ef* at 5 MHz (not shown).

**Figure 4:**
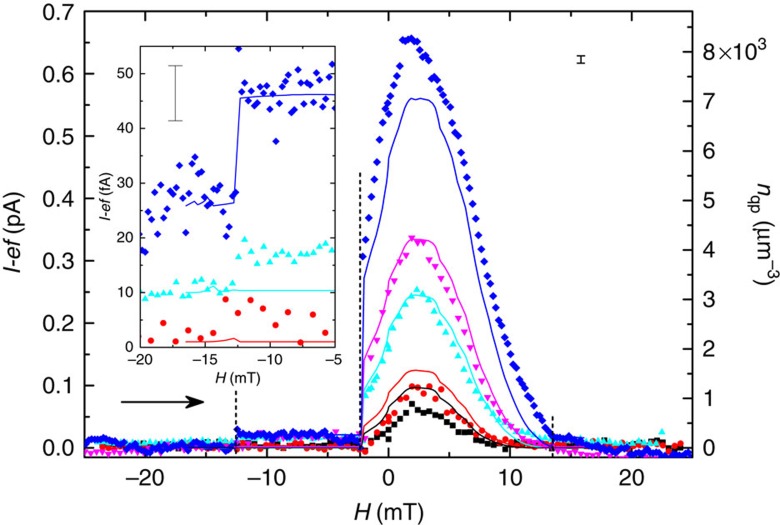
Excess pumping current versus magnetic field. The excess current *I*−*ef* at the first plateau for the driving frequencies 0.5 MHz (black), 1 MHz (red), 5 MHz (cyan), 10 MHz (violet), and 30 MHz (blue) with the maximum electronic temperature at *B*(*H*)=0 of 274, 286, 320, 337 and 369 mK, respectively. Applied magnetic field *H* is swept from −25 to 25 mT (the sweep direction is shown by a horizontal arrow). The vertical dashed lines show the expected values corresponding to the entrance of an extra vortex into the island (for *H*>0) and to the removal of one vortex (*H*<0). (inset) A close-up of the current for a field close to the expulsion of the second vortex. For better visibility, the data sets for different frequencies have been shifted vertically. The measurements were performed at the bias voltage *V*_bias_=100 μV, the gate offset 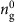
=0.5. In all panels symbols (solid lines) correspond to the experimental data (theoretical model). The scale on the right side shows the QP density *n*_qp_ near the junction. The experimental uncertainty is estimated from the noise of the amplifiers as ∼10 fA, shown as an error bar on both panels.

The turnstile current is expected to assume values equal to integer multiples of *ef* in the absence of non-equilibrium effects and unwanted tunnelling events. The measurements are carried out around the gate offset point 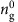
=0.5, to maximize the expected plateau width, for several frequencies *f*. Overheating of the S island, in particular at *H*=2.4 mT corresponding to *B*(*H*)=0, leads to positive deviations of this current from *I*=*nef* (*n* is an integer) by tens percents at the expected plateau positions which corresponds to thousands of quasiparticles per μm^3^ near the junction (see Fig. 3a at *f*=5 MHz). The magnetic field improves quasiparticle trapping: the deviation from *ef* (and the corresponding quasiparticle density) at large enough magnetic fields decreases by an order of magnitude in the whole-frequency range from 0.5 to 200 MHz (see Fig. 4 and Supplementary Note 4) and approaches a few-percent level related to an amplifier noise, even for large gate amplitudes when pumping up to *n*=11 electrons per cycle. The zoom up of the first plateau shown in Fig. 3b demonstrates the magnetic field dependence of *I*. To separate the Meissner current from the vortex contribution, we present in Fig. 3c pumping current versus the field at a fixed gate amplitude value indicated by the vertical dashed line in Fig. 3a. The excess current δ*I*=*I*−*ef* increases when the field is swept from large negative values to low values with jumps at 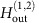 (see vertical dashed lines in Fig. 4). The following field increase to positive values leads to decreasing excess current without visible anomalies. This is related to the difference in the *k*th vortex entry (exit) fields 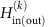. Indeed, for *k*=1 at these fields, we have 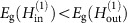, which leads to the efficient redistribution of quasiparticle density even without any vortex (Fig. 1b). Despite the absence of anomalies at the vortex entries, it is possible to estimate the value 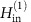 by varying the value of the initial field: the discontinuous anomaly at 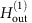 is only visible for a field amplitude in a sweep exceeding 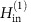
=13.5 mT, which is close to the value found by d.c. measurements in Sample B. At even higher values of the field, 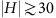 mT, the current quantization is lost again due to the eventual suppression of the S gap near the junctions as well (Supplementary Note 4).

### Theoretical analysis of pumping data

To model theoretically the excess current as a function of the field *B* and frequency *f* we calculate the electronic temperature *T* using a heat balance equation


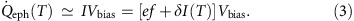


We keep in mind that *T* is nearly uniform and constant in time provided the heat diffusion length *L*_T_ is large compared with the size of the island *R*, and the heat relaxation time 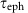, determined by electron–phonon coupling, is much larger than the operating period 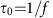, that is, 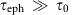 allowing us to average the heat diffusion equation across the sample volume and over the operating period. We assume further that most of the Joule dissipation occurs inside the S island and take into account the excess pumping current *I* averaged over the period from the first plateau on the right hand side (see Supplementary Note 5 for details). The value of 
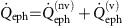
 describes the heat flow rate from the electronic subsystem to phonons for a non-zero depairing parameter Γ. We calculate its value 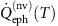 outside the vortex core regions for any Γ combining the procedure described, for example, in refs 38, 39 and the solution of equation (1). For the experimental parameters 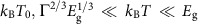 in low-temperature limit the electron–phonon heat flux


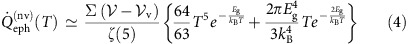


decomposes into recombination 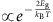 and scattering terms 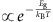 (see, for example, ref. 40). Here Σ is the electron–phonon coupling constant, and 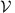 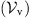 is the volume of the island (the vortex core regions). In the vortex cores in the same limit of negligible phonon temperature 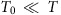 the electron–phonon heat flow is modelled by the standard normal metal expression 
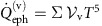
 with the volume of *m* vortex cores assumed to be 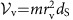. The recombination term in equation (4) becomes dominant at *k*_B_*T*⪞0.1*E*_g_. Beyond the low-temperature limit, we use a numerically calculated expression for 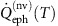 instead of equation (4) (see Supplementary Note 5 for calculation details).

Eventually we obtain the magnetic field and frequency dependence of the measured excess current δ*I*(*T*) as


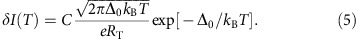


Note that the quasiparticle density near the junction is proportional to the excess current *n*_qp_=*D*(*E*_F_)*eR*_T_δ*I*(*T*)/*C* and can be extracted from δ*I*(*T*) using the normal state density of states in the superconductor *D*(*E*_F_) (see the scale on the right side of Fig. 4 showing the quasiparticle density *n*_qp_). Here *C*∼1 is a numerical coefficient determined by the wave form and the amplitude *A*_g_ of the gate drive, in particular the duration for one junction to be open for tunnelling in each cycle. A detailed derivation is given in Supplementary Note 6. Note that in equation (5) we neglected contributions of higher order processes in 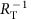 like Andreev tunnelling due to the small transparency of the junctions (see experimental results in ref. 41 and estimates in Supplementary Note 6). By solving (3) with the substituted expressions (2) and (5) we find the solution for *T* and δ*I*(*T*) (solid lines in Fig. 4). We used the constant *C*=1 for a fixed drive amplitude *A*_g_. The main uncertainty in the fitting procedure originates from the parameter 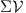. The volume of the S sample can be estimated based on the electron micrograph (Fig. 2b) as 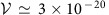 m^3^, but usually this value is overestimated due to additional uncontrolled oxidation of Al. On the other hand, the typical range of the measured values of the electron–phonon relaxation constant Σ in the bulk aluminium^40^,^42^,^43^ is within 2 × 10^8^ to 5 × 10^8^ W K^−5^ m^−3^. Our fitting gives results agreeing reasonably well with the experimental data within the range of 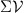 from 4 × 10^−12^ to 9 × 10^−12^ W K^−5^. In Fig. 4, we present a fit for a certain middle value 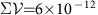 W K^−5^, which is in the best agreement with the experiment at moderate frequencies. Assuming 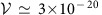 m^3^ we get Σ=2 × 10^8^ W K^−5^ m^−3^, which is towards the low end due to the overestimated 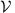 but within the range given above. We have extracted the optimal value of the vortex core radius within the range *r*_v_=2.5–2.7*ξ* both from the d.c. measurements (Supplementary Note 3) and from the pumping data, which is in perfect agreement with the previous theoretical results^19^,^20^.

## Discussion

According to the theoretical model, equation (5), the maximal electronic temperature at *A*_g_=1.1 and *f*=30 MHz is *T*≃370 mK. It corresponds to a number of non-equilibrium quasiparticles 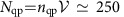 in the uniform state (Fig. 1a). In the field, increasing from *B*(*H*)=0 the Meissner supercurrents sufficiently improve the electron–phonon relaxation by reducing the gap *E*_g_(Γ) in the central part of the island even before the first vortex enters the island. This leads to at least 10–20 times reduction of the quasiparticle density near the junctions when the excess current approaches the amplifier noise level. The vortex contribution is clearly seen in the decreasing field regime due to the hysteresis caused by vortices. Indeed, the vortices that entered the island at a certain value of the field stay there till smaller fields (where the effect of Meissner current is smaller) and improve the relaxation of hot quasiparticles most effectively. Such hysteresis allows us to see the vortex contribution alone (see the larger step in Fig. 4 at *H*∼−2 mT) and the improvement of relaxation in the two-vortex state with respect to the one-vortex state (the smaller step at *H*∼−13 mT). We estimate the recombination rate in the vortex state Γ_rec_≃*f*/*N*_qp,vort_ as the quasiparticle injection rate *f* divided by the quasiparticle number 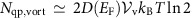 in the vortex core volume 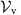 (see Supplementary Note 5 for details). At *f*=30 MHz it gives *N*_qp,vort_∼100, Γ_rec_≃0.3 MHz of the recombination rate, that is, 20 times higher than 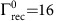 kHz estimated in ref. 40 at *B*=0.

In conclusion, we demonstrate effective control of the number of excess quasiparticles and their spatial distribution in a mesoscopic superconducting disc by applying a small magnetic field on it. We find that both the Meissner supercurrents and vortices entering the disc one by one each give important observable contributions to the trapping of non-equilibrium quasiparticles. We demonstrate that a single-vortex contribution is sufficient to keep the superconducting disc near equilibrium up to 30 MHz injection frequency with *n*_qp_≃400 μm^−3^ quasiparticle density near the junctions and recombination rate of order of Γ_rec_≃0.3 MHz. Our d.c. and pumping measurements confirm the assumption^19^,^20^ that a vortex can be considered as a normal metal cylinder with the effective radius *r*_v_=2.5–2.7*ξ* both in charge and heat transport problems. Our theoretical analysis of the quasiparticle trapping has proven its validity and efficiency in the set-up being in quantitative agreement with the experimental data.

## Methods

### Device fabrication

The hybrid devices with aluminium as the superconductor, copper as the normal metal, and aluminium oxide as the tunnel barrier in between, have been fabricated by standard electron-beam lithography and two-angle shadow evaporation technique. The aluminium island is *d*_S_=20 nm thick and it is oxidized with *O*_2_ for 2 min at 2 mbar. The copper leads, 25 nm thick, are placed on the oxidized Al forming tunnel junctions.

### Sample geometries and parameters

Two different island geometries have been employed in the measurements: Sample B has a nearly square-shaped island, as shown Fig. 2b, and Sample A with the same central part as geometry B has two additional long narrow aluminium extensions from each side towards the junctions (Fig. 2a). The diagonals of the island are 2*R*∼1 μm both in A and B, and the narrow extensions of the island in A are 2 μm long and *w*∼0.13 μm wide. The sum of the tunnel resistances of the two junctions is *R*_T_≃577 kΩ for Sample A and *R*_T_≃714 kΩ for Sample B. We measured the *IV* characteristics of single-electron transistors at various values of the d.c. gate voltage at the base temperature to determine the zero-field S gap value Δ_0_≃190 (207) μeV and the charging energy *E*_C_≃173 (133) μeV for Sample A (B).

### Reproducibility and noise

All the results presented here are reproducible between different runs and between samples of similar geometry, in particular, as concerns the values of the critical fields of vortex entry (exit). The results depend only on whether the absolute field value increases or decreases, provided by the hysteresis in vortex entry/exit events, but they do not depend on the sign of the field as such. The samples are cooled down through the superconducting transition with a zero-field cooled magnet. The uncertainties of current and voltage are estimated to be 10 fA and 3 μV, respectively. They are taken as the s.d. of the signal from the amplifiers.

## Additional information

**How to cite this article:** Taupin, M. *et al*. Tunable quasiparticle trapping in Meissner and vortex states of mesoscopic superconductors. *Nat. Commun.* 7:10977 doi: 10.1038/ncomms10977 (2016).

## Supplementary Material

Supplementary Information Supplementary Notes 1-6 and Supplementary References
