# Supplementary material for: Tunable quasiparticle trapping in Meissner and vortex states of mesoscopic superconductors
Source: Nat Commun. 2016 Mar 16;7:10977. doi: 10.1038/ncomms10977 (PMC4799370; doi:10.1038/ncomms10977)
Supplement: Supplementary Information — Supplementary Notes 1-6 and Supplementary References [file ncomms10977-s1.pdf]

SUPPLEMENTARY NOTE 1. HYSTERESIS IN THE MEASUREMENTS UNDER FIELD

As pointed out in the main text, a remanent field of $\delta H \approx 2.5$ mT and the asymmetry in the Meissner state are present at the sample location at zero applied field $H = 0$ due to the presence of superconducting parts in the sample-holder. The dc measurement presented Fig. 2(d) of the main text for Sample B has been reproduced (on the same sample) in a sample-holder that does not have the superconducting shield for which $B = H$. These measurements are shown in Supplementary Figure 1 for several bias current values $I_{\text{bias}} = 1$ (blue triangles), 10 (red circles), and 100 pA (black squares), together with the theoretical model for the experimental data (solid lines of corresponding colors). The dc voltage V vs field B in this measurement is symmetric with respect to the zero applied field value (except for the vortex hysteresis intrinsic for the sample). The theoretical model presented in Supplementary Note 3 reproduces perfectly the experimental points.

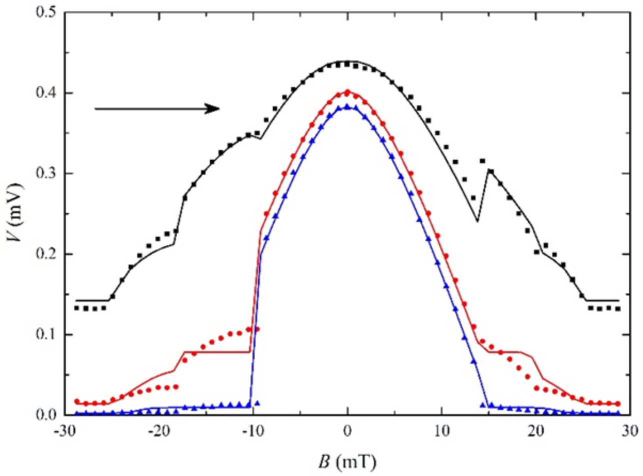

Supplementary Figure 1. **DC measurements without field distortion.** Evolution of the voltage at a fixed bias currents $I_{\text{bias}} = 1$ (blue triangles), 10 (red circles), and 100 pA (black squares) at the gate voltage $C_g V_g/e = n_g = 0.5$ suppressing the Coulomb energy with the magnetic field for Sample B in a sample-holder that does not have the superconducting shield together with the theoretical model (solid lines of corresponding colors). The field B is swept from -30 mT to 30 mT, the direction is shown be the arrow.

In order to fit the theoretical model to our measurements versus field H performed in the sample-holder with the superconducting shield (i.e. with deformation of the field profile), a correction has to be done, as the field H applied through the coil differs from the effective "acting" field B seen by the sample. The field profile has been measured in the shielded sample-holder with a Hall sensor at 4.2 K and at 0.2 K. The first measurement has been done in the normal state when there is no magnetic shielding as a reference point (not shown). The second measurement of the effective field B versus the applied coil magnetic field H swept from -30 mT to 30 mT (at 0.2 K) is shown in Supplementary Figure 2 as black dashed lines. The arrows point out the direction of the sweep. For $|H| > 20$ mT, the sample-holder is fully normal and the effective field equals the applied one. For $|H| < 20$ mT, a nonlinear superconducting response from the sample holder is present leading to hysteresis. The red line in Supplementary Figure 2 is the correction found by comparing directly the dc measurements in both sample-holders ($V(H)$ in Fig. 2(d) of the main text and $V(B)$ in Supplementary Figure 1). The two methods yield very similar results. Eventually theoretical curves given as functions of B by the model are presented as functions of H using red curve $B(H)$ in Supplementary Figure 2.

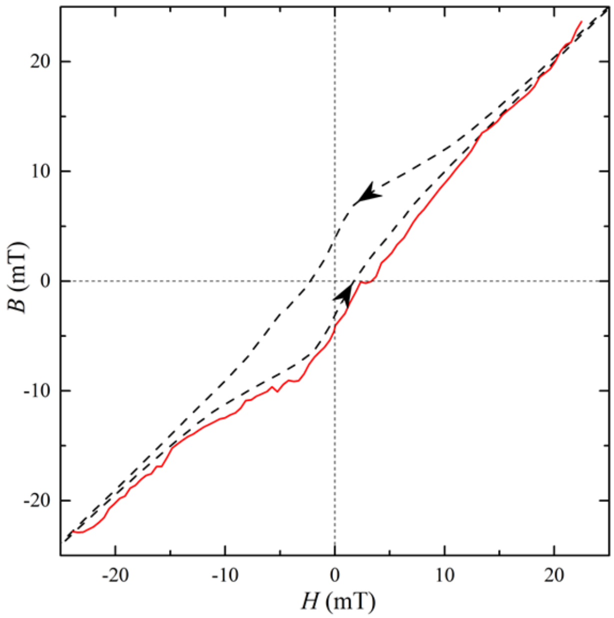

Supplementary Figure 2. **Magnetization curves.** Field correction found by using a Hall sensor (black dashed lines) and by comparing the dc measurements of $V(H)$ in both sample holders (red solid line). The largest deviation from linearity lies at low field. The two methods yield a very similar correction.

SUPPLEMENTARY NOTE 2. HOMOGENEOUS APPROXIMATION IN A MESOSCOPIC SAMPLE

The samples studied experimentally are in the dirty regime, namely $l \ll \xi$, where $\xi = \sqrt{\hbar D/\Delta_0}$ the coherence length, D is the diffusion coefficient, l is the elastic mean free path, and Δ_0 is the superconducting gap. The quasiparticle (QP) spectral characteristics in this case can be found from the Usadel equations (see, e.g., Ref. [1])

$$\frac{\hbar D}{2}\nabla^2\theta(\boldsymbol{r}) + \left(iE - \frac{\hbar}{2D}\boldsymbol{v}_S^2\cos\theta(\boldsymbol{r})\right)\sin\theta(\boldsymbol{r}) + \Delta\cos\theta(\boldsymbol{r}) = 0, \qquad \mathrm{div}\,(sin^2\theta(\boldsymbol{r})\boldsymbol{v}_S) = 0, \quad (1)$$

where $g^R = -g^{R*} = \cos\theta(r)$ and $f^R = -f^{A*} = -i\sin\theta(\mathbf{r})$ are the normal and anomalous Green's functions (superscripts 'R' and 'A' stand for 'retarded' and 'advanced').

Considering the experimental situation of a mesoscopic superconducting sample with the characteristic size R = 0.5 μm and the coherence length $\xi \sim 100-200$ nm (depending on the diffusion coefficient), we have to verify if we can neglect the gradient terms in Sup. Eq. (1). For subgap energies the characteristic length scale of the function $\theta(\mathbf{r})$ can be estimated as follows: $\xi/\sqrt{1 - E^2/\Delta^2}$. It is natural to assume the $\theta(\mathbf{r})$ function inhomogeneity to be small provided $\xi/\sqrt{1 - E^2/\Delta^2} > R$. This condition gives us the energy interval $1 - E^2/\Delta^2 < \xi^2/R^2 \sim 0.1$, sufficient for the calculations of the electron-phonon heat flow $\dot{Q}_{\mathrm{eph}}$ and the thermal excitation leakage current δI for temperatures much lower than the superconducting gap. Indeed, the main contribution to $\dot{Q}_{\mathrm{eph}}$ and to δI is given by $|E/\Delta - 1| \sim k_B T/\Delta \simeq 0.03 < \xi^2/R^2 \simeq 0.1$.

Certainly the above assumption is strictly valid only for the Meissner state: inside the vortex core the gap and the anomalous Green function turn to zero at the scale of the effective core radius rv which is of order of the coherence length ξ [2].

To avoid numerical solution of the Usadel equation we adopt in the main text the following approximate procedure. In the presence of vortices we assume that both the order parameter and $\theta(\mathbf{r})$ function vanish inside the vortex cores while outside the core regions we assume the $\theta(\mathbf{r})$ function to vary slowly and introduce, thus, its average θ over the region outside the vortex cores (omitting the spatial dependence in the notation). The deviations from the averaged order parameter Δ beyond the cores also become small in this limit. Integrating now the above Usadel equation over the region outside the vortex cores we obtain Eq. (1) from the main text with the effective depairing parameter expressed through the superfluid velocity **vs** as

$$\Gamma = \frac{\hbar}{2D}\langle \mathbf{v}_S^2\rangle = \frac{\hbar D}{2}\langle(\nabla\varphi - 2e\mathbf{A}/\hbar c)^2\rangle. \quad (2)$$

Here the brackets $\langle .. \rangle$ denote an average over the sample volume (over the central part of the sample A) with the excluded vortex core regions, φ is the superconducting order parameter phase and **A** is the vector potential determined by the external magnetic field **B** applied to the sample. The second Usadel equation in our approximation reduces to div $\mathbf{v}_s = 0$ and leads to the vanishing components of **vs** perpendicular to the sample boundary and to the boundaries of vortex cores. Here and further on we neglect the changes in the magnetic field B due to the screening currents flowing in the sample because of the smallness of the characteristic sample size R as compared to the effective screening length $\lambda_{\mathrm{eff}} = \lambda^2/d_S$. For our samples $\lambda \simeq 230$ nm [4] and d_S = 20 nm, therefore $\lambda_{\mathrm{eff}} \simeq 2.6$ μm.

Solution of the averaged Usadel equation gives us the expression for the hard gap E_g in the density of states and for the order parameter Δ as functions of Γ as[1,5-7]

$$E_g = \Delta\,(1 - \gamma^{2/3})^{3/2}\,,\ \Delta = \Delta_0 e^{-\pi\gamma/4}\,,\ \gamma = \Gamma/\Delta\,. \quad (3)$$

In the main text we focus on the case $\gamma < 1$ ($\Gamma < \Delta_0 e^{-\pi/4}$), implying that the gap $E_g > 0$ is non-zero.

SUPPLEMENTARY NOTE 3. DC FITTING

Using the solution of the averaged Usadel equation, Eq. (1) from the main text, one can fit the *IV* characteristics shown in Supplementary Figure 1. Indeed, we consider a hybrid single electron transistor (SET), namely, a mesoscopic superconducting island tunnel coupled to the normal metal leads (NISIN). We apply a fixed bias current $I_{\rm bias}$ through the normal leads and the constant gate voltage $n_{\rm g} = C_{\rm g} V_{\rm g}/e = 0.5$ to the gate electrode coupled to the island through the capacitor $C_{\rm g}$ (see black and red lines in Fig. 2(c) of the main text) and measure the difference V of voltages $V_{\rm L,R} = \pm V/2$ applied to the leads as a function of the magnetic field B seen by the sample.

In stationary state the current $I_{\rm bias}$ flowing from one lead to another is equal in any cross section and it can be calculated in any of two junctions (for example, in the left one)

$$I_{\rm bias} = -e\sum_k p_k\left[\Gamma^{\rm L}_{k\to k+1}(V) - \Gamma^{\rm L}_{k\to k-1}(V)\right] \tag{4}$$

as a sum over the island charge state k of the sequential tunneling rates $\Gamma^{\rm L}_{k\to k+1}$ ($\Gamma^{\rm L}_{k\to k-1}$) to (from) the island through the left junction. This sum is weighted with the probability p_k of system being in this charge state, which is calculated using the standard rate equation for the balance of the probability fluxes[8-10]

$$\frac{dp_k}{dt} = \sum\left[\Gamma_{k\pm1\to k}p_{k\pm1} - \Gamma_{k\to k\pm1}p_k\right], \qquad \sum p_k = 1\,, \tag{5}$$

in the stationary case $dp_k/dt = 0$ with the tunneling rate $\Gamma_{k\to k\pm1} = \sum_{i={\rm L,R}}\Gamma^i_{k\to k\pm1}$ and $\Gamma^i_{k\to k\pm1} = \Gamma\left[U^\pm_{k,i}\right]$ given by

$$\Gamma[U] = \frac{2}{e^2R_{\rm T}}\int n_{\rm S}(E)f_T(E)\left[1 - f_{T_0}(E+U)dE\right] \tag{6}$$

Here $U^\pm_{k,i} = \mp 2E_{\rm C}(k - n_{\rm g} \pm 1/2) \mp eV_i$ are the energies gained by the electrons tunneling to/from the island (being in the charge state k) through ith junction, $R_{\rm T}/2$ is the tunnel resistance of each junction. Here we focus on the magnetic field effects in the sample B (see Fig. 2(b) in the main text) and neglect all the overheating effects assuming the equilibrium Fermi distribution of electrons over energy $fT(E) = [e^{E/k_{\rm B}T} + 1]-1$ with the electron temperature T equal to the phonon bath temperature T_0. The density of states (DOS) $n_{\rm S}(E) = {\rm Re}[\cos\theta]$ normalized to its normal state value $D(E_{\rm F})$ in the superconducting (S) island near the junction is obtained from the solution of averaged Usadel equation, Eq.(1) from the main text, with the depairing parameter $\Gamma/\Delta_0 = \alpha_1(B/B_{\rm c})^2$- $m\alpha_2 B/B_{\rm c} + m^2\alpha_3$, Eq. (2) in the main text, having three positive numerical fitting parameters α_l.

In the Sample B the tails of the wave functions localized in the vortex core(s) give a substantial contribution to the DOS and to *IV* curves for $m \neq 0$ at small $I_{\rm bias}$, but they are not included into the averaged model. To model this contribution we replace the DOS $n_{\rm S}(E)$ by $n_{\rm S}(E)\,(1 - e^{-R/r_{\rm v}}) + e^{-R/r_{\rm v}}$ by adding the phenomenological normal metal DOS with the exponentially suppressed prefactor $e^{-R/r_{\rm v}}$ determined by the vortex distance from the junction $R \sim 0.5\ \mu$m and by the exponential decay of the wave function localized in the vortex core of the effective radius $r_{\rm v}$. This vortex contribution leads to reduced $V(B)$ at small $I_{\rm bias}$ in the mixed state $m \neq 0$ and to the suppression of the jumps at the vortex entry fields (see red and blue curves in Supplementary Figure 1).

By fitting $V(B)$ at $I_{\rm bias} = 100$ pA which is not affected by the vortex tail contributions one can extract the following values of fitting parameters $\alpha_1 = 0.38$, $\alpha_2 = 0.438$, and $\alpha_3 = 0.266$ mentioned in the main text. Following [11] we attribute to all jumps in this plot with the change of the number of vortices in the sample and use the point of the first jump at $B > 0$ as the field of the first vortex entry $B_{\rm C} = 14.4$ mT. In this setup we don't see any transitions between vortex configurations with the constant vorticity like the transition to a giant vortex state (see, e.g., [12, 13]). Using these parameters one can fit $V(B)$ quite well at all bias current values with $R/r_{\rm v} \sim 1.7$. The optimal value of the vortex core radius $r_{\rm v} = 2.5 - 2.7\xi$ extracted from dc measurements in the Sample B is in perfect agreement with the previous theoretical works[2,3]. In subgap regime $I_{\rm bias} = 1$ and 10 pA the jump-like anomalies in $V(B)$ become knee-like, but because of the above-mentioned reasons we still associate each of them with the vortex entry or exit.

SUPPLEMENTARY NOTE 4. ELECTRONIC PUMPING

The electronic pumping of the Sample B at f = 5 MHz, when $V_{bias} \approx 120\ \mu$V and $n_g \sim 0.5$, is shown in Supplementary Figure 3(a) with the field H swept from −10 mT to 2 mT. Contrary to what is observed in the Sample A, the increase of the magnetic field increases the deviation from the current quantization $I = ef$, due to the effect of the screening current on the superconducting gap. This observation is in agreement with the theoretical model with the increasing number of QPs in S island with the field.

The electronic pumping of the Sample A at frequency f=200 MHz, when $V_{bias} \approx 250\ \mu$V and $n_g \sim 0.5$, is shown in Supplementary Figure 3(b) for two fields values H = 0 and −20 mT. Similarly to the lower frequency range the increase of the magnetic field reduces the QP contribution to the excess current.

The evolution of the pumping current in the Sample A with the field is shown in Supplementary Figure 4. In panel (a) we show the pumping current versus field with the different initial field values. For a small value of the initial field H = −12 mT, the island is in the Meissner state and a continuous variation of current is observed. The anomaly at −2 mT appears only if the initial field is large enough to turn island into the mixed state (see blue and red curves). Similarly, the anomaly at $H_{out}^{(2)} \sim -15$ mT (see inset of Fig. 4 of main text) appears only if the initial field exceeds 20 mT. Panel (b) shows that the current deviates significantly from $I = ef$ at field larger than ∼30 mT due to the reduction of the S gap near the junctions.

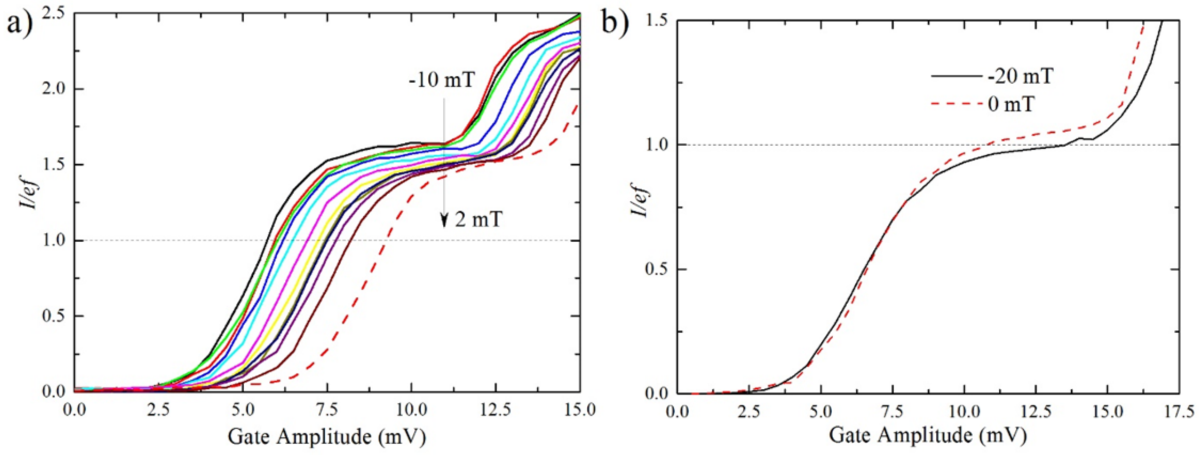

Supplementary Figure 3. **Pumping of Sample B and sample A at 200 MHz.** (a) Electronic pumping of the Sample B at f = 5 MHz at fixed bias voltage V_{bias} = 120 μV under field H from−10 mT to 2 mT (from top to bottom). With the field, the deviation from $I = ef$ increases rather than decreases in contrast to the behavior of Sample A. (b) Electronic pumping of the Sample A at f = 200 MHz at fixed bias voltage V_{bias} = 250 μV in field H = 0 (red dashed line) and H = −20 mT (solid black line).

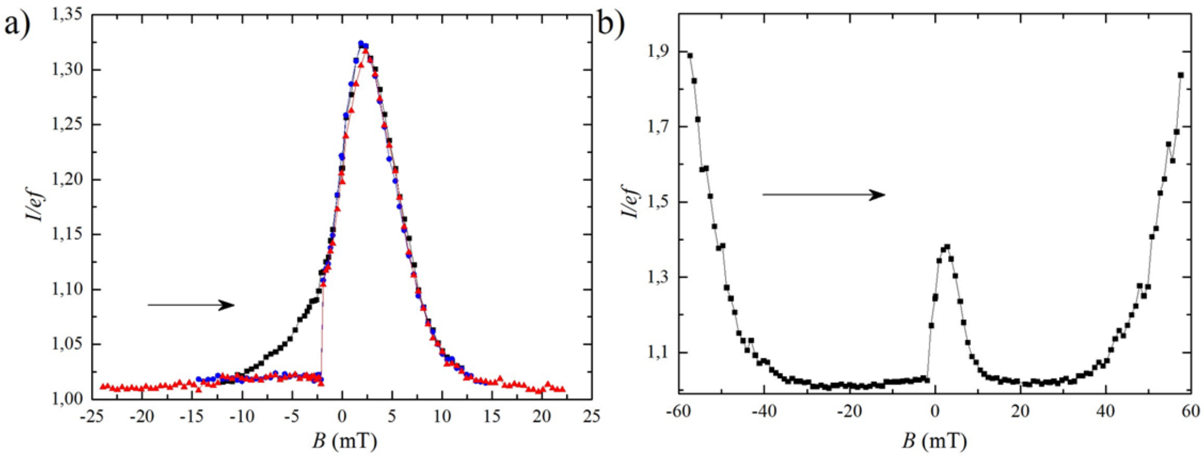

Supplementary Figure 4. **Extra pumping measurements of Sample A.** (a) Evolution of the electronic pumping with the magnetic field measured in the range ±12 mT (black squares), ±15 mT (blue open cirles) and ±25 mT (red crosses). At the initial field value the island is in a vortex free, in a single-vortex, or in a two-vortex state, respectively. (b) Pumping current in a wide field range, from −60 mT to 60 mT. The pumping has been measured with the gate offset n_g^{0} = 0.5 and the bias voltage V_{bias}=100 μV at f = 5 MHz.

SUPPLEMENTARY NOTE 5. HEAT BALANCE EQUATION

In this section we describe the theoretical model of the relaxation of QPs in applied magnetic field by using the example of NISIN SET in the turnstile regime. By applying the constant bias voltages $V_{L,R} = \pm V_{bias}/2$ to the normal leads and the periodic gate voltage $n_g(t) = C_g V_g(t)/e = n_g^0 + A_g \sin(2\pi f t)$ with a certain offset n_g^0, frequency f, and the amplitude A_g to the gate electrode one can push electrons to tunnel through the system producing a time-dependent current $I(t)$. This transport current $I(t)$ flowing from one lead to another drives the NISIN turnstile out of the equilibrium by injecting nonequilibrium QPs into the S island. The power $\dot{Q}_{\rm NIS}^{\rm S}$ injected to the island increases with the frequency f and we model this increase in mean density of QPs in the superconductor by raising its electron temperature T relatively to the phonon bath temperature T_0. Note that the quasiequilibrium Fermi distribution of electrons over energy $f_T(E) = [e^{E/k_B T} + 1]^{-1}$ is provided by the smallness of the inelastic electron-electron scattering time τ_{ee} comparing to the operating time $\tau_0 = 1/f$ and the effective charging time e/I.[14]

Due to the large electron-phonon relaxation length $L_{\rm T} \gg R$ compared to the island size R we consider the heat balance equation[4] for the whole island

$$\dot{Q}_{\rm NIS}^{\rm S} = \dot{Q}_{\rm eph}, \tag{7}$$

where $\dot{Q}_{\rm eph}$ is the electron-phonon heat flow averaged over the island volume $\mathcal{V}$. The power injected into the island from the junction can be written as follows

$$\dot{Q}_{\rm NIS}^{\rm S} = \sum_{k,i} p_k\left[\dot{Q}_{\rm S}(U_{k,i}^{+}) + \dot{Q}_{\rm S}(U_{k,i}^{-})\right] \tag{8}$$

as a sum of the power dissipated in the S island in a single tunneling event

$$\dot{Q}_{\rm S}(U) = \frac{1}{e^2 R_{\rm T}} \int E_{\rm S} n_{\rm S}(E) f_T(E)\left[1 - f_{T_0}(E+U)\right] dE \tag{9}$$

over the junction i = L, R through which electron tunnels and over the island charge state k. This sum is weighted with the probability p_k of system being in this charge state, which is calculated using the standard rate equation for the balance of the probability fluxes given by Sup. Eq. (5). Here $E_{\rm S} = E$. Note that here and further on we neglect the difference between the electronic temperature $T_{\rm N}$ in the normal metal of volume $\mathcal{V}_{\rm N}$ and the bath temperature T_0, because of sufficient electron-phonon relaxation there $Q_{\rm eph}^{\rm N} = \Sigma_{\rm N}\mathcal{V}_{\rm N}\left(T_{\rm N}^5 - T_0^5\right)$ ($\Sigma_{\rm N}$ the electron-phonon material constant) and the rather small power $\dot{Q}_{\rm NIS}^{\rm N}$ injected into the normal leads which can be obtained from Sup. Eq. (8) by replacing $E_{\rm S}$ by $E_{\rm N} = eV_i - E$ in Sup. Eq. (9).

Due to the Joule's law the sum of the powers injected into the island $\dot{Q}_{\rm NIS}^{\rm S}$ and into the normal leads $\dot{Q}_{\rm NIS}^{\rm N}$ should be equal to IV, where I is the current in Sup. Eq. (4) averaged over the period $\tau_0 = 1/f$ of the gate voltage operation. Usually if the superconductor is not overheated much $k_{\rm B}T \ll E_{\rm g}$ one can assume that $\dot{Q}_{\rm NIS}^{\rm S} \gg \dot{Q}_{\rm NIS}^{\rm N}$ in subgap regime and $\dot{Q}_{\rm NIS}^{\rm S} \simeq \dot{Q}_{\rm NIS}^{\rm N}$ at biases above the gap[14]. As a result within constant factor of order of unity the heat balance equation can be rewritten as follows

$$\dot{Q}_{\rm eph} \simeq IV. \tag{10}$$

Within the optimal conditions of the proper turnstile shielding and optimized device geometry the averaged current I is close to its ideal value ef and the deviation $\delta I = I - ef$ is mainly governed by nonequilibrium QP density in the S island near the junction

$$n_{\rm qp}(T) = 2D(E_{\rm F})\int_0^\infty n_{\rm S}(E) f_T(E)\, dE \approx D(E_{\rm F})\sqrt{2\pi k_{\rm B} T \Delta_0}\, e^{-\Delta_0/k_{\rm B}T}. \tag{11}$$

The estimates for contributions in higher orders in small parameter $\hbar/e^2 R_{\rm T}$ are given in the following Note.

The latter expression is written for the sample A where the S gap near the junction is close to Δ_0. At $B = 0$ the QP number N_{qp} in the S island equals $N_{\rm qp} = n_{\rm qp}\mathcal{V}$ the product of n_{qp} to the volume of the island $\mathcal{V}$. The QP number $N_{\rm qp,vort} \simeq 2D(E_{\rm F})\mathcal{V}_{\rm v}k_{\rm B}T \ln 2$ in the vortex core can be obtained from Sup. Eq. (11) by substitution of the normal state DOS $n_S(E) = 1$ into the integral and by multiplying it by the vortex core volume $\mathcal{V}_{\rm v} = r_{\rm v}^2 d_{\rm S} \sim 7\xi^2 d_{\rm S}$.

The electron-phonon heat flow under magnetic field is similar to the expression given by Eq. (3) in[15]

$$\dot{Q}_{\rm eph} = \frac{\Sigma\mathcal{V}}{24\zeta(5)k_{\rm B}^5}\int_0^\infty \epsilon^3\left[n_T(\epsilon) - n_{T_0}(\epsilon)\right]d\epsilon\int_{-\infty}^{\infty} M_{E,E+\epsilon}[f(E) - f(E+\epsilon)]dE. \qquad (12)$$

with the term $n_{\rm S}(E)n_{\rm S}(E+\epsilon)[1 - \Delta_0^2/E(E+\epsilon)]$ substituted by $M_{E,E+\epsilon}$ of the form[16]

$$8M_{E,E'} = 2(g_{E'}^{\rm R} - g_{E'}^{\rm A})(g_E^{\rm R} - g_E^{\rm A}) - (f_{E'}^{\rm R} - f_{E'}^{\rm A})(f_E^{\dagger\rm R} - f_E^{\dagger\rm A}) - (f_E^{\rm R} - f_E^{\rm A})(f_{E'}^{\dagger\rm R} - f_{E'}^{\dagger\rm A}). \qquad (13)$$

Here Σ is the electron-phonon material constant, and $\zeta(s)$ is the Riemann zeta function. The retarded (advanced) normal $g^{\rm R(A)}$ and anomalous $f^{\rm R(A)}$ Green's functions are determined by the solution of Eq. (1) from the main text, i.e., $M_{E,E'} = n_{\rm S}(E)n_{\rm S}(E') - b(E)b(E')$, with $n_S(E) = {\rm Re}[\cos\theta]$ and $b(E) = {\rm Im}[\sin\theta]$.

In the low temperature limit T_0, $T \ll E_{\rm g}/k_{\rm B}$ the main contribution to Sup. Eq. (12) arises from the energies $0 < |E| - E_{\rm g} \lesssim k_{\rm B}T$ close to the hard gap value $\pm E_{\rm g}$, which can be calculated using the following expansion of $n_S(E)$ and $b(E)$ over the small positive parameter $\delta E = |E| - E_{\rm g} \ll E_{\rm g}$

$$n_{\rm S}(E)^2 = \Theta(\delta E)\frac{2\delta E\Delta^{2/3}}{3\Gamma^{4/3}E_{\rm g}^{1/3}}, \qquad \frac{b(E)}{n_{\rm S}(E){\rm sign}(E)} \approx \left(\frac{E_{\rm g}}{\Delta}\right)^{1/3} \qquad (14)$$

Here $\Theta(x)$ is the Heaviside theta-function.

Substituting Sup. Eqs. (13, 14) into Sup. Eq. (12) and taking into account only the leading terms in the small parameter $k_B T/E_g$ we obtain

$$\dot{Q}_{\rm eph} = \frac{\Sigma\mathcal{V}}{\zeta(5)}\left\{\frac{64}{63}T^5 e^{-\frac{E_{\rm g}}{k_{\rm B}T}} + \frac{2\pi E_{\rm g}^4}{3k_{\rm B}^4}e^{-\frac{2E_{\rm g}}{k_{\rm B}T}}\right\} \qquad (15)$$

for rather large electronic temperatures $\Gamma^{2/3}E_{\rm g}^{1/3}/k_{\rm B}, T_0 \ll T \ll E_{\rm g}/k_{\rm B}$. Note that the recombination term ($\propto e^{-2E_{\rm g}/k_{\rm B}T}$) dominates at $k_B T > 0.1E_g$ and should be taken into account. In the opposite case $T_0 \ll T \ll \Gamma^{2/3}E_{\rm g}^{1/3}/k_{\rm B}$

$$\dot{Q}_{\rm eph} = \frac{\Sigma\mathcal{V}T^3}{9\zeta(5)\Gamma^{2/3}E_{\rm g}^{1/3}}\left\{\frac{128}{21}k_{\rm B}T^3 e^{-\frac{E_{\rm g}}{k_{\rm B}T}} + \frac{\pi E_{\rm g}^3}{k_{\rm B}^2}e^{-\frac{2E_{\rm g}}{k_{\rm B}T}}\right\} \qquad (16)$$

Here the recombination term is of order of the scattering term ($\propto e^{-E_{\rm g}/k_{\rm B}T}$) at $k_B T \sim 0.3 E_g$. In both cases as the temperature becomes of the order of the gap one have to use full numerical expression given by Sup. Eq. (12).

SUPPLEMENTARY NOTE 6. EXCESS CURRENT AS A FUNCTION OF ELECTRONIC TEMPERATURE

To calculate our main observable, the leakage current $\delta I = I - ef$ in the NISIN turnstile we use the simplified version of the master equation given in Sup. Eq. (5) for low temperatures taking into account only two charge states $k = 0$ and $k = 1$

$$\frac{dp_1}{dt} = \Gamma_{0\to1}p_0 - \Gamma_{1\to0}p_1, \quad p_0 = 1 - p_1 \tag{17}$$

with the tunneling rates given in Sup. Eq. (6) in the subgap regime $\left|U_{0,i}^{+}\right| < E_{\mathrm{g}}$ given by

$$\Gamma[U] \approx \Gamma_{T_0} e^{-(E_{\mathrm{g}}-U)/k_{\mathrm{B}}T_0} + \Gamma_T e^{-E_{\mathrm{g}}/k_{\mathrm{B}}T} \tag{18}$$

Here $U_{0,i}^{+} = -U_{1,i}^{-} = 2E_{\mathrm{C}}A_{\mathrm{g}}\sin(2\pi f t) - eV_i$ and $\Gamma_0 = \sqrt{2\pi k_{\mathrm{B}}TE_{\mathrm{g}}}/e^2R_{\mathrm{T}}$. This expression contains the exponentially growing part with U which determines the dominant tunneling rate with maximal U for each time instant.

We consider the offset $n_{\mathrm{g}}^{0} = 0.5$ for simplicity and use the symmetry of the drive $n_{\mathrm{g}}(\tau_0 - t) = 1 - n_{\mathrm{g}}(t)$ focusing on the first half of the period with n_{g} increasing from $0.5 - A_{\mathrm{g}}$ to $0.5 + A_{\mathrm{g}}$. We assume that before the time instant t_1 the island is discharged $k = 0$ due to the domination rate $\Gamma_{1\to0}^{\mathrm{R}}$ among the others and the charging process is started at $t = t_1$. The probability $P_0(t)$ to stay in the state $k = 0$ is decreasing with time $t > t_1$ as

$$P_0(t) = \exp\left[-\int_{t_1}^{t}\Gamma_{0\to1}(t')dt'\right]. \tag{19}$$

For typical frequencies the charging process occurs not far from $n_{\mathrm{g}} = 0.5$, therefore further we linearize the drive $n_{\mathrm{g}}(t) \approx 0.5 + 2\pi A_{\mathrm{g}}(ft - 1/4)$. As the island has been charged $P_0(t^*) = \epsilon \lesssim 1$ (let's take $\epsilon = 1/2$ for definiteness) the leakage current starts to flow. The number of excess electrons N_{l} through the island can be written as the integral of the largest subleading rate $\Gamma_{1\to0}^{\mathrm{R}}(t)$ governing the leakage current over the time interval $t^* < t < t_2$ before this rate becomes the dominant one

$$N_{\mathrm{l}} \simeq \int_{t^*}^{t_2}\Gamma_{1\to0}^{\mathrm{R}}(t)dt. \tag{20}$$

The leakage current can be calculated as follows $\delta I \simeq 2efN_{\mathrm{l}}$, where “2” accounts for the leakage during the second half of the period due to the symmetry $k \leftrightarrow 1-k$ and L $\leftrightarrow$ R.

By substituting Sup. Eq. (18) in Sup. Eqs. (19, 20) and calculating the time instants $t_1 = \tau_0 - t_2$ and t^* one can come to the result

$$\delta I = I - ef \simeq e\Gamma_T\left[1 - \frac{2E_{\mathrm{g}} - |e|V - k_{\mathrm{B}}T_0 a(T)}{2\pi E_{\mathrm{C}}A_{\mathrm{g}}}\right]e^{-\frac{E_{\mathrm{g}}}{k_{\mathrm{B}}T}} \tag{21}$$

where $a = \ln\frac{\Gamma_{T_0}k_{\mathrm{B}}T_0}{2\pi E_{\mathrm{C}}A_{\mathrm{g}}f\ln2}$ for $n_{\mathrm{g}}(t_1) = n_{\mathrm{g}}(t_2) = 0.5$ at low enough electronic temperature $T \lesssim T_0\left[1 - (eV/2E_{\mathrm{g}})\right]^{-1}$ and $a(T) = |e|V/2 - \frac{E_{\mathrm{g}}}{k_{\mathrm{B}}}(1 - T_0/\mathrm{T}) + \ln\frac{\Gamma_{T_0}k_{\mathrm{B}}T_0{}^2}{2\pi E_{\mathrm{C}}A_{\mathrm{g}}fT\ln2}$ for $n_{\mathrm{g}}(t_1) = 1 - n_{\mathrm{g}}(t_2) < 0.5$ in the opposite case $T \gtrsim T_0\left[1 - (eV/2E_{\mathrm{g}})\right]^{-1}$. In this derivation we consider the operating frequency f to be small compared to the charging rate $\sim (t^* - t_1)^{-1}$ to avoid missing events. We neglect the relative corrections of order of $e^{-e|V|/k_{\mathrm{B}}T_0}$ ($\Gamma_{0\to1}^{\mathrm{R}}/\Gamma_{0\to1}^{\mathrm{L}}$ and $\Gamma_{1\to0}^{\mathrm{L}}/\Gamma_{1\to0}^{\mathrm{R}}$ for the case when the first term in Sup. Eq. (18) dominates for all rates). We don't take into account the factor ½ in N_{l} during the time when $\Gamma_{0\to1}^{\mathrm{L}} \gg$

$\Gamma^{\mathrm{L}}_{1\to 0} = \Gamma^{\mathrm{R}}_{1\to 0} \simeq \Gamma_T e^{-E_{\mathrm{g}}/k_{\mathrm{B}}T}$, when the discharging occurs with the equal probability $p_{\mathrm{L,R}} = 1/2$ to left and to the right contact. We can do it, because during the integration of Sup. Eq. (20) $U^-_{1,\mathrm{L}}$ can go beyond the subgap range $U^-_{1,\mathrm{L}} < -E_{\mathrm{g}}$ suppressing the second term in Sup. Eq. (18) for $\Gamma^{\mathrm{L}}_{1\to 0}$ exponentially $\sim e^{-(E_{\mathrm{g}}+|U^-_{1,\mathrm{L}}|)/k_{\mathrm{B}}T}$ and keeping the rate $\Gamma^{\mathrm{R}}_{1\to 0}$ to be the dominant one in the leakage current.

To avoid all these unimportant details we consider a certain A_{g} -dependent numerical prefactor $C \sim 1$ instead of the square brackets in Sup. Eq. (21) and come to Eq. (5) of the main text by using the assumption that the S gap near the junction (in the sample A) is close to Δ_0.

Comparing Sup. Eq. (11) and Eq. (5) in the main text one can write down the following relation between the leakage current $\delta I = I - ef$ and the QP density n_{qp} near the junctions

$$n_{\mathrm{qp}} = D(E_{\mathrm{F}})eR_{\mathrm{T}}\delta I/C \qquad (22)$$

used in Fig. 4 of the main text to show the QP density scale.

Note that we neglect also the contributions of higher orders in the small parameter $\hbar/(e^2R_{\mathrm{T}})$ like Andreev tunneling (see, e.g., [17, 18]) due to rather large tunnel resistance of the sample contacts. Indeed, from the experimental side the attribute feature of Andreev tunneling is the additional peak in the beginning of each current plateau $I = nef$[17] which is not observed in all pumping measurements of this paper. From the theoretical side one can estimate the relative contribution $\delta I_{\mathrm{AR}}/(ef)$ of Andreev tunneling to the current as the ratio $\Gamma_{\mathrm{AR}}/\Gamma[U]$ of dc rates of sequential $\Gamma[U] = U/(e^2R_{\mathrm{T}})$ and Andreev tunneling $\Gamma_{\mathrm{AR}} \simeq \pi\hbar U/(4Ne^4R_{\mathrm{T}}^2)$ in the above-gap regime. Here $N = A/A_{\mathrm{ch}}$ is the number of channels in the tunnel junction, $A \simeq 6\cdot 10^3\ \mathrm{nm}^2$ is the area of the junction and A_{ch} is the area of a single channel. Theoretical estimates given in [18] lead to $A_{\mathrm{ch}} \sim 2\ \mathrm{nm}^2$, while experimental observation[17] gives $A_{\mathrm{ch}} \sim 30\ \mathrm{nm}^2$. The upper bound estimate with $A_{\mathrm{ch}} \sim 30\ \mathrm{nm}^2$ and $R_{\mathrm{T}} = 577\ \mathrm{k\Omega}$ for the sample A gives $N \sim 200$ and $\delta I_{\mathrm{AR}}/(ef) \sim \pi\hbar/(4Ne^2R_{\mathrm{T}}) \simeq 3.10^{-5}$ which can be neglected comparing to the QP contribution.

SUPPLEMENTARY REFERENCES

[1] Anthore, A., Pothier, H. & Esteve, D. Density of states in a superconductor carrying a supercurrent. *Phys. Rev. Lett.* **90,** 127001 (2003).

[2] Golubov, A. A. & Houwman, E. P. Quasiparticle relaxation rates in a spatially inhomogeneous superconductor. *Physica C: Superconductivity* **205,** 147-153 (1993).

[3] Golubov, A. A. *et al.* Quasiparticle lifetimes and tunneling times in a superconductor-insulator-superconductor tunnel junction with spatially inhomogeneous electrodes. *Phys. Rev. B* **49,** 12953-12968 (1994).

[4] Peltonen, J. T., Muhonen, J. T., Meschke, M., Kopnin, N. B. & Pekola, J. P. Magnetic-field-induced stabilization of nonequilibrium superconductivity in a normal- metal/insulator/superconductor junction. *Phys. Rev. B* **84,** 220502(R) (2011).

[5] Skalski, S., Betbeder-Matibet, O. & Weiss, P. R. Properties of superconducting alloys containing paramagnetic impurities. *Phys. Rev.* **136,** A1500-A1518 (1964).

[6] Maki, K. & Fulde, P. Equivalence of different pair-breaking mechanisms in superconductors. *Phys. Rev.* **140,** A1586-A1592 (1965).

[7] Fulde, P. Tunneling density of states for a superconductor carrying a current. *Phys. Rev.* **137,** A783-A787 (1965).

[8] Averin, D. & Likharev, K. Coulomb blockade of single-electron tunneling, and coherent oscillations in small tunnel junctions. *Journal of Low Temperature Physics* **62,** 345-373 (1986).

[9] Fulton, T. A. & Dolan, G. J. Observation of single-electron charging effects in small tunnel junctions. *Phys. Rev. Lett.* **59,** 109-112 (1987).

[10] Likharev, K. Single-electron transistors: electrostatic analogs of the DC SQUIDS. *IEEE Trans. Magnetics* **23,** 1142-1145 (1987).

[11] Kanda, A., Baelus, B. J., Peeters, F. M., Kadowaki, K. & Ootuka, Y. (2004). Experimental evidence for giant vortex states in a mesoscopic superconducting disk. *Phys. Rev. Lett.* **93,** 257002 (2004).

[12] Schweigert, V. A., Peeters, F. M. & Singha Deo, P. Vortex phase diagram for mesoscopic superconducting disks. *Phys. Rev. Lett.* **81,** 2783-2786 (1998).

[13] Palacios, J. J. Vortex matter in superconducting mesoscopic disks: Structure, magnetization, and phase transitions. *Phys. Rev. B* **58,** R5948-R5951(R) (1998)

[14] Giazotto, F., Heikkilä, T. T., Luukanen, A., Savin, A. M. & Pekola, J. P. Opportunities for mesoscopics in thermometry and refrigeration: Physics and applications. *Rev. Mod. Phys.* **78,** 217 - 274 (2006).

[15] Maisi, V. F. *el al.* Excitation of single quasiparticles in a small superconducting Al island connected to normal-metal leads by tunnel junctions. *Phys. Rev. Lett.* **111,** 147001 (2013).

[16] Kopnin, N. B. *Theory of Nonequilibrium Superconductivity*. (Oxford Univ. Press, Oxford, 2001).

[17] Aref, T. *el al.* Andreev tunneling in charge pumping with SINIS turnstiles. *Europhys. Lett.* **96,** 37008 (2011).

[18] Averin, D. V. & Pekola, J. P. Nonadiabatic Charge Pumping in a Hybrid Single-Electron Transistor. *Phys. Rev. Lett.* **101,** 066801 (2008).
